# Characterization of patients with adrenal insufficiency and frequent adrenal crises

**DOI:** 10.1530/EJE-20-1324

**Published:** 2021-03-25

**Authors:** Marcus Quinkler, Robert D Murray, Pinggao Zhang, Claudio Marelli, Robert Petermann, Andrea M Isidori, Bertil Ekman, the EU-AIR Investigators

**Affiliations:** 1 Endocrinology in Charlottenburg, Berlin, Germany; 2 Department of Endocrinology, Leeds Teaching Hospitals NHS Trust, St James’s University Hospital, Leeds, UK; 3 Shire Human Genetic Therapies, a member of the Takeda Group of Companies, Cambridge, Massachusetts, USA; 4 Shire International GmbH, a member of the Takeda Group of Companies, Zug, Switzerland; 5 Baxalta Innovations GmbH, a member of the Takeda Group of Companies, Vienna, Austria; 6 Department of Experimental Medicine, Sapienza University of Rome, Rome, Italy; 7 Departments of Endocrinology in Linköping, and Health, Medicine and Caring Sciences, Linköping University, Linköping, Sweden

## Abstract

**Objective:**

This study aimed to characterize the clinical and biochemical features of patients with primary (PAI) and secondary (SAI) adrenal insufficiency who developed adrenal crises (ACs) and estimate the incidence of ACs in these patients.

**Design:**

Retrospective case-control analysis of the European Adrenal Insufficiency Registry (EU-AIR; NCT01661387).

**Methods:**

Two thousand six hundred and ninety-four patients with AI (1054 PAI; 1640 SAI) enrolled in EU-AIR. Patients who developed ≥ 1 AC were matchd 1:3 with patients without ACs for age, sex and AI type. Data were collected at baseline and follow-up (mean ± s.d.: PAI 3.2 ± 1.7 years; SAI 2.9 ± 1.7 years).

**Results:**

One hundred and forty-eight out of 2694 patients (5.5%; *n*  = 84 PAI; *n*  = 64 SAI) had an AC during the study: 6.53 (PAI) and 3.17 (SAI) ACs/100 patient-years. Of patients who experienced an AC, 16% (PAI) and 9.4% (SAI) experienced ≥ 1 AC/year. The incidence of adverse events, infectious intercurrent illnesses and infectious serious adverse events were higher in patients with ACs than without ACs. No differences were observed in BMI, HbA_1c_, blood pressure and frequencies of diabetes mellitus or hypertension between subgroups (PAI and SAI, with and without ACs). At baseline, PAI patients with AC had higher serum potassium (4.3 ± 0.5 vs 4.2 ± 0.4 mmol/L; *P* = 0.03) and lower sodium (138.5 ± 3.4 vs 139.7 ± 2.9 mmol/L; *P* = 0.004) than patients without AC. At last observation, SAI patients with AC had higher hydrocortisone doses than patients without AC (11.9 ± 5.1 vs 10.1 ± 2.9 mg/m^2^; *P* < 0.001).

**Conclusions:**

These results demonstrate that concomitant diseases and cardiovascular risk factors do not feature in the risk profile of AC; however, patients with AC had a higher incidence of infectious events.

## Introduction

Since the introduction of synthetic glucocorticoid replacement therapy, the life expectancy of adequately treated patients with adrenal insufficiency (AI) has been considered normal (1, 2). In recent years, however, patients with AI have been shown to have increased mortality and morbidity as well as impaired quality of life (3). Increased mortality and morbidity vs the general population have been shown in patients with AI in general (4), and in both primary AI (PAI) (5, 6) and secondary AI (SAI) (7) individually. There are several aspects of the disease and its management that have been proposed to contribute to these observations. First, a putative increase in cardiovascular risk associated with higher glucocorticoid replacement doses (8, 9, 10); second, a high rate of infections in these individuals, leading to more frequent hospital admissions (11); and third, inadequate glucocorticoid dose escalation in emergency situations, which may contribute to the observed increased mortality (12).

A significant proportion of patients with AI experience adrenal crises (ACs) and require hospital admission (13). In retrospective studies, the incidence of ACs has been reported to be 5.2–17 per 100 patient-years in PAI, and 3.6–5.8 per 100 patient-years in SAI (13, 14, 15). The only prospective study to date, which investigated ACs over a period of 2 years, confirmed these estimates (16). This study reported an incidence of 8.3 ACs per 100 patient-years, with the ACs being fatal in 6.3% of cases (16).

Infectious diseases, particularly gastroenteritis, are reported to be the most common precipitating factors for ACs (13, 14, 16, 17). Emotional stress, missed glucocorticoid doses or inadequate glucocorticoid dose increases may also be the factors facilitating ACs (16, 18). Several retrospective studies have attempted to identify risk factors for ACs. Data from the UK Addison’s Disease Self-Help Group indicated that concomitant diseases such as diabetes, asthma or premature ovarian failure are associated with an increased incidence of ACs (17). A further study confirmed that concomitant non-endocrine disease and age at diagnosis were risk factors for ACs in PAI, and diabetes insipidus was a risk factor in SAI (13). In a third study, risk factors for ACs were identified as cardiac, neurological and pulmonary comorbidities as well as malignancy (14). However, in a subgroup of patients with autoimmune PAI, there was no increased risk of AC in the presence of other associated autoimmune diseases such as diabetes mellitus type 1, thyroid disease, coeliac disease, vitiligo or autoimmune premature menopause (14). The only prospective study reported that patients with a previous history of AC at baseline analysis had a significantly higher risk of AC during follow-up than patients without a previous history of AC (16).

Many of the controversies relating to the frequency of ACs and their predisposing factors result from differences between studies in which patients with AI are not actively participating and those studies with a prospective setting and recruitment. Considering the lack of clarity regarding precipitating factors for ACs in AI, the primary objective of this registry-based analysis of real-world data was to characterize patients who developed ACs during a prospective observational period. The secondary objective was to compare clinical and biochemical data of patients with PAI and SAI who have ACs with those from patients with no ACs, to identify potential risk factors.

## Methods

### Study population

The European Adrenal Insufficiency Registry (EU-AIR) is an observational, open-ended study (ClinicalTrials.gov identifier: NCT01661387) of patients with PAI, SAI or congenital adrenal hyperplasia (CAH) who are receiving long-term treatment with modified-release hydrocortisone or other glucocorticoid replacement therapies (19, 20). The primary objective of EU-AIR is to monitor the safety of long-term treatment with once-daily, modified-release hydrocortisone and other glucocorticoid replacement therapies in patients with AI.

Clinical and biochemical data from the time of study enrolment (baseline) to the date of the last follow-up visit were collected prospectively. Data collected included patient demographics and characteristics, relevant medical history and concomitant diseases, type of glucocorticoid replacement therapy, concomitant medications, vital signs and laboratory assessments. Episodes of ACs and other adverse events (AEs) were recorded. An AC was defined as an acute impairment of general health with the need for parenteral hydrocortisone and saline infusion (21). The occurrence of ACs was determined as the number of events per patient group, and the incidence rate as the number of events/100 patient-years in each group. An AE was defined as any untoward, undesired or unplanned clinical event in the form of signs, symptoms, disease, or laboratory or physiological observations occurring in a patient participating in the clinical study, regardless of causal relationship to treatment. It was the responsibility of the EU-AIR physician to determine if an AE or AC event had occurred, and to record the duration of the event and dosing change in the electronic case report form.

All enrolled patients provided written informed consent/assent for inclusion in the registry. Data were collected from endocrinology centres in Germany, Italy, the Netherlands, Sweden and the United Kingdom. The study protocol was approved by the appropriate ethics committee in each country (Ethic committee at Gothenburg University, Gothenburg, Sweden, Ref. No: 721-12; NRES Committee Yorkshire & the Humber – Humber Bridge, UK, Ref No: 12/YH/0313; Ethic Committee of the Charité University Medicine Berlin, Germany, Ref. No: EA2/101/12; Ethic Committee of the Azienda Ospedaliero Universitaria Policlinico Umberto, Italy, Ref. No: I-RIF/CE/4562; Code Committee and the Appeals Committee of the Pharmaceutical Advertising Code Foundation, Stichting Code Geneesmiddelen Reclame (CGR), Amsterdam, Netherlands, Ref.No: ADVICE A14.027). Patients were followed prospectively during the course of routine clinical practice for the active duration of the registry. All medical care decisions, including those relating to treatment choice, were entirely at the discretion of the patient and registry physician. Patient data, including laboratory assessments, were collected using an electronic case report form at enrolment and thereafter at routine clinic visits (every 6–12 months).

Recruitment of patients commenced in August 2012. As of February 2019, a total of 2694 patients with AI (PAI, *n* = 1054; SAI, *n* = 1640) had been enrolled in EU-AIR.

For this analysis, patients with CAH were not included. We chose a nested case–control study design, and patients were grouped according to type of AI (PAI or SAI) and whether they had experienced an AC during the study period (August 2012 to February 2019). Each of the patients in the registry who experienced an AC during the study period were matched with patients who had not experienced an AC and were in the same age group (10–40, >40–60, >60–80 or >80 years old); the same sex (male or female) and same AI type (PAI or SAI). In the matching process, if there were ≥3 patients with no ACs who were of the same age group, sex, and AI type, three of these patients were selected at random. The patient who had an AC and the three selected patients with no ACs were called matched patients and were included in the analyses. If there were <3 patients without ACs of the same age group, sex, and AI type as a patient who had an AC, the patient who had experienced an AC was not included in the analyses.

### Statistical analysis

Statistics for continuous variables were primarily descriptive: mean, standard deviation (s.d.), median and range. Incidence and percentage, as well as incidence based on patient-years, were used for categorical variables. Differences were calculated for continuous variables and odds ratios and 95% CIs were calculated for categorical variables between the ACs and no ACs subgroups. *P* values were from Student's *t*-test for continuous variables or *Χ^2^* test for categorical variables. *P* values are used to indicate trends rather than hypothesis testing.

## Results

### Adrenal crises

The overall EU-AIR cohort comprised 1054 patients with PAI and 1640 with SAI. In total, 168 ACs were documented in 1054 patients with PAI (2572 patient-years) and 113 ACs were reported in 1640 patients with SAI (3547 patient-years). These data translated to an incidence of 6.53 ACs per 100 patient-years in PAI, and 3.17 ACs per 100 patient-years in SAI.

**Figure 1:**
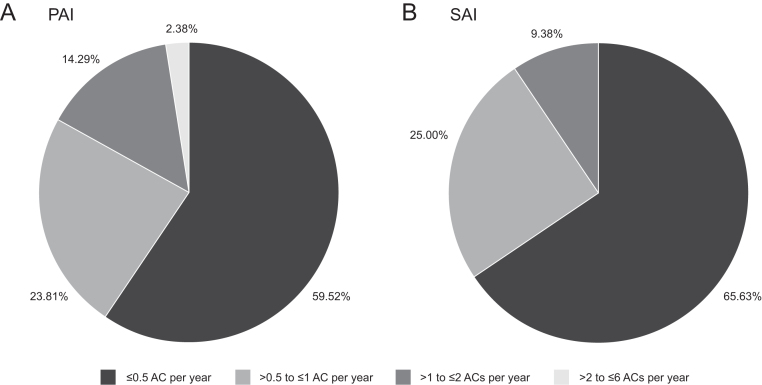
Frequency of ACs in patients with (A) PAI (*n* = 84) and (B) SAI (*n* = 64) with frequent ACs.The frequency of ACs was calculated as the number of ACs per year of observation. The total may be greater than 100% owing to rounding. AC, adrenal crisis; PAI, primary adrenal insufficiency; SAI, secondary adrenal insufficiency.

During the study period, ≥1 AC was documented in 148 patients: 84 with PAI and 64 with SAI. Nearly 60% of the patients with PAI who experienced ≥ 1 AC had a low incidence of < 0.5 ACs/year (Fig. 1A); however, 40% of patients with PAI experienced ≥ 1 AC every second year. Among the latter, more than 16% had ≥ 1 AC per year (Fig. 1A). In patients with SAI with ACs, 65% had a low incidence of < 0.5 ACs/year and only 9.4% experienced ≥ 1 AC/year (Fig. 1B).

### Characterization of patients with adrenal crises

#### Patient selection and baseline demographics

The 148 patients who experienced ≥ 1 AC were matched 1:3 with a comparator group of patients who had no ACs. This latter cohort comprised 252 patients with PAI and 192 with SAI. Baseline patient demographics and characteristics are provided in Table 1. Patient groups were well matched, with the only differences occurring in duration of disease. Patients with PAI with ACs had a significantly shorter mean ± s.d. duration of disease (11.9 ± 11.3 vs 15.8 ± 12.9 years; *P* = 0.013) than patients without ACs (Table 1). The opposite was observed between the two subgroups of patients with SAI: patients with ACs had a significantly longer mean duration of disease than patients with no ACs (14.2 ± 12.0 vs 10.8 ± 10.0 years; *P* = 0.026).

**Table 1:** Baseline demographics and comorbidities of patients with PAI and SAI. Patients with PAI and SAI who experienced ≥ 1 AC during the study period until the data cut-off point of May 2, 2019 were categorized as ‘≥ 1 AC’ (*n* = 84, *n* = 64, respectively). Those two groups were matched 1:3 for age, sex and AI type with patients with AI and no ACs, labelled ‘no AC’ (*n* = 252, *n* = 192, respectively).

	PAI				SAI			
	≥1 AC (*n* = 84)	No AC (*n* = 252)	OR (95% CI)	*P* value	≥1 AC (*n* = 64)	No AC (*n* = 192)	OR (95% CI)	*P* value
Female sex, *n* (%)	66 (78.6)	198 (78.6)	–	1.000	39 (60.9)	117 (60.9)	–	1.000
Age at enrolment, y	–	–	–	0.661	–	–	–	0.672
Mean ± s.d.	49.1 ± 15.9	49.9 ± 15.7	–	–	53.4 ± 15.9	52.4 ± 16.0	–	–
Median (range)	48.5 (21–84)	51.0 (18–88)	–	–	55.0 (18–85)	53.0 (19–86)	–	–
Duration of disease, y	–	–	–	0.013	–	–	–	0.026
Mean ± s.d.	11.9 ± 11.3	15.8 ± 12.9	*–*	–	14.2 ± 12.0	10.8 ± 10.0	–	–
Median (range)	8.5 (0–52.2)	12.9 (0–70.3)	–	–	12.5 (0.3–42.9)	7.6 (0–41.9)	–	–
Type of diagnosis, *n* (%)	–	–	–	–	–	–	–	–
Metabolic syndrome	13 (15.5)	38 (15.1)	1.0 (0.5–2.0)	0.930	15 (23.4)	39 (20.3)	1.2 (0.6–2.4)	0.596
Hypertension	18 (21.4)	50 (19.8)	1.1 (0.6–2.0)	0.754	20 (31.3)	67 (34.9)	0.9 (0.5–1.6)	0.594
DM	17 (20.2)	42 (16.7)	1.3 (0.7–2.4)	0.456	8(12.5)	24 (12.5)	1.0 (0.4–2.4)	1.000
Type 1	8 (9.5)	15 (6.0)	1.7 (0.7–4.1)	0.262	0 (0)	2 (1.0)	0	1.000
Type 2	8 (9.5)	21 (8.3)	1.2 (0.5–2.7)	0.737	5 (7.8)	16 (8.3)	0.9 (0.3–2.7)	0.895
Dyslipidaemia	14 (16.7)	46 (18.3)	0.9 (0.5–1.7)	0.742	12 (18.8)	54 (28.1)	0.6 (0.3–1.2)	0.138

AC, adrenal crisis; AI, adrenal insufficiency; DM, diabetes mellitus; OR, odds ratio; PAI, primary AI; SAI, secondary AI.

#### Treatment of AI

The median duration of follow-up in each of the four patient subgroups was between 2.5 and 4.1 years. At baseline and last observation, there were no differences in the daily dose of glucocorticoid per body surface area between patients with PAI with ACs and those without ACs, irrespective of the type of glucocorticoid administered (Table 2). In patients with SAI with ACs, however, the mean daily hydrocortisone dose per body surface area at last observation was significantly higher than in patients without ACs (11.9 ± 5.1 vs 10.1 ± 2.9 mg/m^2^; *P <* 0.001) (Table 3).

**Table 2:** Clinical and laboratory data of patients with PAI at baseline and last observation. Patients with PAI who experienced ≥ 1 AC during the study period until the data cut-off point of February 5, 2019 were categorized as ‘≥ 1 AC’ (*n* = 84). This group was matched 1:3 for age, sex and AI type with patients with AI and no ACs, labelled ‘no AC’ (*n* = 252).

	Baseline			Last observation		
	≥1 AC (*n* = 84)	No AC (*n* = 252)	*P* value	≥1 AC (*n* = 84)	No AC (*n* = 252)	*P* value
Duration of follow-up, y	–	–	–	–	–	–
Mean ± s.d.	–	–	*–*	3.83 ± 1.44	2.92 ± 1.73	–
Median (range)	–	–	–	4.08 (0.96–6.17)	2.50 (0.005–6.17)	–
Type of glucocorticoid, *n* (%)	–	–	–	–	–	–
Cortisone acetate	1 (1.2)	7 (2.8)	–	1 (1.2)	6 (2.4)	–
Hydrocortisone	62 (73.8)	184 (73.0)	–	58 (69.0)	131 (52.0)	–
Modified-release hydrocortisone	18 (21.4)	43 (17.1)	–	17 (20.2)	26 (10.3)	–
Prednisolone	2 (2.4)	15 (6.0)	–	2 (2.4)	12 (4.8)	–
Daily dose (mg)	–	–	–	–	–	–
Cortisone acetate	35.0	29.3 ± 12.3	–	35.0	34.2 ± 5.6	–
Hydrocortisone	23.3 ± 7.2	22.8 ± 7.2	0.604	21.2 ± 7.5	22.3 ± 9.2	0.398
Modified-release hydrocortisone	23.9 ± 4.0	24.5 ± 5.5	0.657	22.9 ± 4.7	25.4 ± 5.5	0.138
Prednisolone	5.3 ± 3.2	6.1 ± 3.0	0.715	5.0 ± 0	5.3 ± 0.9	0.339
Daily dose per BSA (mg/m^2^) g	–	–	–	–	–	–
Cortisone acetate	19.0	18.6 ± 3.6	––	18.6	19.2 ± 3.1	–
Hydrocortisone	13.1 ± 4.0	12.5 ± 4.3	0.349	11.8 ± 4.3	12.1 ± 5.3	0.639
Modified-release hydrocortisone	13.0 ± 2.3	13.9 ±2.7	0.227	12.4 ± 3.1	13.9 ± 2.5	0.094
Prednisolone	3.2 ± 2.1	3.8 ± 1.6	0.679	3.1 ± 0.1	3.0 ± 0.5	0.665
Fludrocortisone daily dose (mg)	0.1 ± 0.07	0.1 ± 0.1	0.827	0.1 ± 0.07	0.1 ± 0.07	0.333
DHEA therapy	–	–	–	–	–	–
*n* (%)	14 (16.7)	14 (5.6)	0.003	15 (17.9)	15 (6.0)	0.002
OR (95% CI)	–	–	3.4 (1.5–7.5)	–	–	3.4 (1.6–7.4)
BMI (kg/m^2^)	–	–	0.375	–	–	0.252
Mean ± s.d.	25.5 ± 4.7	26.1 ± 5.1	–	25.8 ± 4.8	26.6 ± 6.3	–
Median (range)	24.6 (18.7–40.8)	25.1 (18.6–42.5)	–	24.9 (16.7–40.6)	25.6 (11.5–56.7)	–
Waist circumference (cm)	–	–	0.904	–	–	0.646
Mean ± s.d.	90.9 ± 11.4	91.2 ± 13.2	–	88.6 ± 14.4	89.2 ± 14.8	–
Median (range)	90.0 (72–121)	89.0 (70–132)	–	87.0 (57–134)	88.0 (62–132)	–
Systolic blood pressure (mmHg)	–	–	0.125	–	–	0.068
Mean ± s.d.	121.9 ± 20.6	125.7 ± 18.6	–	123.9 ± 19.7	128.5 ± 19.5	–
Median (range)	118.0 (80–195)	122.5 (80–197)	–	120.0 (90–197)	126.0 (80–197)	–
Diastolic blood pressure (mmHg)	–	–	0.284	–	–	0.016
Mean ± s.d.	76.1 ± 9.2	77.5 ± 10.4	–	75.4 ± 9.7	78.5 ± 10.5	–
Median (range)	77.5 (50–100)	79.0 (50–110)	–	75.0 (58–114)	80.0 (50–104)	–
Biochemical parameters, mean ± s.d.	–	–	–	–	–	–
Serum potassium (mmol/L)	4.3 ± 0.5	4.2 ± 0.4	0.031	4.4 ± 0.4	4.3 ± 0.4	0.145
Serum sodium (mmol/L)	138.5 ± 3.4	139.7 ± 2.9	0.004	139.5 ± 2.8	139.8 ± 2.9	0.327
Triglycerides (mmol/L)	–	–	0.899	–	–	0.829
Mean ± s.d.	1.6 ± 0.9	1.6 ± 0.9	–	1.6 ± 0.9	1.6 ± 0.9	–
Median (range)	1.3 (0.4–4.7)	1.3 (0.5–5.2)	–	1.3 (0.4–5.1)	1.3 (0.5–5.4)	–
Cholesterol (mmol/L)	–	–	0.125	–	–	0.085
Mean ± s.d.	5.3 ± 1.1	5.5 ± 1.0	–	5.2 ± 1.0	5.4 ± 1.1	–
Median (range)	5.1 (3.4–8.1)	5.5 (3.0–9.0)	–	5.0 (3.3–8.1)	5.4 (2.7–9.0)	–
HDL-cholesterol (mmol/L)	–	–	0.144	–	–	0.073
Mean ± s.d.	1.7 ± 0.5	1.8 ± 0.5	–	1.6 ± 0.5	1.8 ± 0.5	–
Median (range)	1.6 (0.8–2.7)	1.7 (0.6–3.0)	–	1.6 (0.9–2.7)	1.7 (0.6–3.4)	–
LDL-cholesterol (mmol/L)	–	–	0.310	–	–	0.144
Mean ± s.d.	3.1 ± 1.0	3.3 ± 0.9	–	2.9 ± 0.8	3.1 ± 0.9	–
Median (range)	3.1 (1.3–6.0)	3.2 (1.2–5.8)	–	2.9 (1.4–5.0)	3.0 (1.0–5.5)	–
HbA_1c_ (%)	–	–	0.185	–	–	0.187
Mean ± s.d.	5.8 ± 0.9	5.6 ± 0.8	–	5.1 ± 1.4	4.9 ± 1.4	–
Median (range)	5.6 (4.6–8.7)	5.4 (4.5–10.0)	–	5.3 (2.6–9.6)	5.1 (2.0–10.0)	–
Statin therapy	–	–	0.724	–	–	0.592
*n* (%)	11 (13.1)	38 (15.1)	–	14 (16.7)	35 (13.9)	–
OR (95% CI)	–	–	0.8 (0.4–1.7)	–	–	1.2 (0.6–2.4)

AC, adrenal crisis; AI, adrenal insufficiency; BSA, body surface area; HDL, high-density lipoprotein; LDL, low-density lipoprotein; OR, odds ratio; PAI, primary AI.

**Table 3:** Clinical and laboratory data of patients with SAI at baseline and last observation. Patients with SAI who experienced ≥ 1 AC during the study period until the data cut-off point of February 5, 2019 were categorized as ‘≥ 1 AC’ (*n* = 64). This group was matched 1:3 for age, sex and AI type with patients with AI and no ACs, labelled ‘no AC’ (*n* = 192).

	Baseline			Last observation		
	≥1 AC (*n* = 64)	No AC (*n* = 192)	*P* value	≥1 AC (*n* = 64)	No AC (*n* = 192)	*P* value
Duration of follow-up, y	–	–	–	–	–	0.015
Mean ± s.d.	–	–	*–*	–	3.48 ± 1.64	2.86 ± 1.68
Median (range)	–	–	–	–	3.78 (0.22–6.04)	2.88 (0.02–6.24)
Type of glucocorticoid, *n* (%)	–	–	–	–	–	–
Cortisone acetate	2 (3.1)	7 (3.6)	–	2 (3.1)	4 (2.1)	–
Hydrocortisone	50 (78.1)	167 (87.0)	–	45 (70.3)	110 (57.3)	–
Modified-release hydrocortisone	4 (6.3)	13 (6.8)	–	4 (6.3)	7 (3.6)	–
Prednisolone	7 (10.9)	4 (2.1)	–	6 (9.4)	4 (2.1)	–
Daily dose (mg)	–	–	–	–	–	–
Cortisone acetate	14.4 ± 6.2	23.9 ± 9.6	0.234	15.0 ± 7.1	26.3 ± 2.5	0.035
Hydrocortisone	19.7 ± 3.5	20.4 ± 6.8	0.343	23.3 ± 10.0	19.5 ± 6.1	<0.001
Modified-release hydrocortisone	22.5 ± 2.9	22.3 ± 8.3	0.965	22.5 ± 5.0	24.3 ± 9.3	0.734
Prednisolone	4.6 ± 1.8	4.9 ± 0.3	–	4.1 ± 1.4	4.4 ± 1.0	0.731
Daily dose per BSA (mg/m^2^) g	–	–	–	–	–	–
Cortisone acetate	8.8 ± 4.7	15.1 ± 3.3	0.091	9.5 ± 5.2	14.3 ± 2.2	0.159
Hydrocortisone	10.3 ± 2.4	10.7 ± 3.5	0.396	11.9 ± 5.1	10.1 ± 2.9	<0.001
Modified-release hydrocortisone	10.7 ± 1.4	10.8 ± 1.7	0.920	10.6 ± 1.1	11.1 ± 1.4	0.583
Prednisolone	2.6 ± 1.3	2.5 ± 0.1	0.878	2.2 ± 0.9	2.2 ± 0.5	0.988
DHEA therapy	–	–	–	–	–	–
*n* (%)	5 (7.8)	4 (2.1)	0.046	7 (10.9)	8 (4.2)	0.063
OR (95% CI)	–	–	4.0 (1.0–15.3)	–	–	2.8 (1.0–8.1)
BMI (kg/m^2^)	–	–	0.697	–	–	0.915
Mean ± s.d.	28.6 ± 5.5	29.0 ± 5.1	–	29.8 ± 6.6	29.7 ± 5.9	–
Median (range)	27.8 (19.2–42.6)	28.4 (18.9–45.0)	–	28.0 (20.2–48.4)	29.1 (16.2–49.7)	–
Waist circumference (cm)	–	–	0.700	–	–	0.485
Mean ± s.d.	99.0 ± 14.1	97.9 ± 13.3	–	99.4 ± 14.4	97.7 ± 12.6	–
Median (range)	98.0 (75–129.5)	98.0 (70–133)	–	98.0 (72–130.5)	97.0 (66.5–133)	–
Systolic blood pressure (mmHg)	–	–	0.292	–	–	0.072
Mean ± s.d.	129.2 ± 21.5	132.4 ± 20.4	–	128.3 ± 18.0	133.5 ± 20.5	–
Median (range)	127.0 (90–200)	130.0 (80–197)	–	124 (98–192)	130 (97–197)	–
Diastolic blood pressure (mmHg)	–	–	0.730	–	–	0.328
Mean ± s.d.	79.7 ± 11.6	80.3 ± 11.5	–	79.0 ± 10.3	80.6 ± 11.4	–
Median (range)	80.0 (54–110)	80.0 (51–128)	–	80.0 (53–110)	80.0 (51–128)	–
Biochemical parameters, mean ± s.d.	–	–	–	–	–	–
Serum potassium (mmol/L)	4.1 ± 0.3	4.2 ± 0.4	0.176	4.2 ± 0.3	4.2 ± 0.4	0.518
Serum sodium (mmol/L)	140.0 ± 3.2	140.8 ± 2.9	0.144	140.0 ± 3.1	140.7 ± 3.2	0.185
Triglycerides (mmol/L)	–	–	0.436	–	–	0.801
Mean ± s.d.	1.8 ± 0.8	1.9 ± 1.0	–	1.9 ± 0.9	2.0 ± 1.0	–
Median (range)	1.8 (0.6–4.4)	1.7 (0.5–5.9)	–	1.7 (0.6–4.3)	1.7 (0.5–5.1)	–
Cholesterol (mmol/L)	–	–	0.127	–	–	0.760
Mean ± s.d.	5.6 ± 1.0	5.3 ± 1.1	–	5.3 ± 1.1	5.2 ± 1.2	–
Median (range)	5.4 (3.6–7.6)	5.1 (2.3–8.8)	–	5.4 (2.1–8.2)	5.2 (2.5–9.2)	–
HDL-cholesterol (mmol/L)	–	–	0.720	–	–	0.281
Mean ± s.d.	1.5 ± 0.5	1.5 ± 0.5	–	1.4 ± 0.4	1.4 ± 0.5	–
Median (range)	1.4 (0.6–3.0)	1.4 (0.7–2.9)	–	1.3 (0.8–2.2)	1.3 (0.6–2.7)	–
LDL-cholesterol (mmol/L)	–	–	0.084	–	–	0.460
Mean ± s.d.	3.6 ± 1.0	3.2 ± 1.0	–	3.2 ± 0.9	3.1 ± 1.1	–
Median (range)	3.5 (1.6–5.3)	3.1 (1.3–7.2)	–	3.1 (1.0–5.8)	3.0 (0.5–7.1)	–
HbA_1c_ (%)	–	–	0.579	–	–	0.273
Mean ± s.d.	5.5 ± 0.5	5.6 ± 0.7	–	5.0 ± 1.4	4.7 ± 1.2	–
Median (range)	5.5(4.6–6.9)	5.4 (4.6–8.1)	–	5.3 (2.3–9.4)	5.1 (2.5–8.6)	–
Statin therapy	–	–	0.724	–	–	0.592
*n* (%)	6 (9.4)	43 (22.4)	0.027	7 (10.9)	36 (18.8)	0.179
OR (95% CI)	–	–	0.4 (0.1–0.9)	–	–	0.5 (0.2–1.3)

AC, adrenal crisis; AI, adrenal insufficiency; BSA, body surface area; HDL, high-density lipoprotein; LDL, low-density lipoprotein; OR, odds ratio; PAI, primary AI.

At baseline, patients with SAI with ACs had a higher frequency of receiving DHEA therapy than those without ACs (7.8% vs 2.1%; *P* = 0.046) (Table 3). Similarly, patients with PAI with ACs had a higher frequency of DHEA therapy at baseline (16.7% vs 5.6%; *P* = 0.003) and at last observation (17.9% vs 6.0%; *P* = 0.002) compared with those without ACs (Table 2). In patients with PAI, no difference in fludrocortisone dose was found between the two subgroups at baseline or last observation.

#### Comorbidities and clinical measures

When examining the frequency of comorbidities within our subgroups, there were no differences in the frequency of diagnosis of metabolic syndrome, hypertension or diabetes mellitus between patients with PAI with or without ACs (Table 1). Likewise, no differences in the frequency of diagnosis of hypothyroidism or premature ovarian failure were detected between the two PAI groups (Table 4). No differences in the frequency of diagnosis of any of the concomitant diseases studied were found between the two subgroups of patients with SAI (Tables 1 and 4). In patients with SAI with ACs, the frequency of statin therapy at baseline was significantly lower than in patients without ACs (9.4% vs 22.4%; *P* = 0.027) (Table 3). This was not observed at last observation and was not reflected in any of the lipid parameters measured.

**Table 4:** Disease-specific comorbidities of patients with PAI and SAI. Patients with PAI and SAI who experienced ≥ 1 AC during the study period until the data cut-off point of February 5, 2019 were categorized as ‘≥ 1 AC’ (*n* = 84, *n* = 64, respectively). Those two groups were matched 1:3 for age, sex and AI type with patients with AI and no ACs, labelled ‘no AC’ (*n* = 252, *n* = 192, respectively). Data are presented as *n* (%).

	≥1 AC	No AC	OR (95% CI)	*P* value
PAI	–	–	–	–
* n*	84	252	–	–
* *Diagnosis of hypothyroidism	27 (32.1)	54 (21.4)	1.7 (1.0–3.0)	0.061
* *Diagnosis of premature ovarian failure	2 (2.4)	2 (0.8)	3.0 (0.4–22.0)	0.247
SAI	–	–	–	–
* n*	64	192	–	–
* *Diagnosis of diabetes insipidus	10 (15.6)	31 (16.1)	1.0 (0.4–2.1)	0.843

AC, adrenal crisis; AI, adrenal insufficiency; OR, odds ratio; PAI, primary AI; SAI, secondary AI.

At baseline and at last observation, no differences were found between the subgroups with and without ACs regarding systolic blood pressure, concentrations of triglycerides, cholesterol, lowdensity lipoprotein-cholesterol (LDL), high-density lipoprotein-cholesterol (HDL) or HbAl. Patients with PAI with ACs had significantly higher mean serum potassium (4.3 ± 0.5 vs 4.2 ± 0.4 mmol/L; *P* = 0.03) and lower serum sodium (138.5 ± 3.4 vs 139.7 ± 2.9 mmol/L; *P* = 0.004) at baseline than patients without ACs (Table 2). At last observation, no difference was found in these two parameters in patients with PAI, but diastolic blood pressure was lower (75.4 ± 9.7 vs 78.5 ± 10.5 mmHg; *P* = 0.016) in patients with ACs than in patients without ACs (Table 2).

#### Adverse events

The 15 most frequently reported AEs were nasopharyngitis, pyrexia, fatigue, diarrhoea, nausea, vomiting, headache, gastrointestinal infection (gastroenteritis), influenza, upper respiratory infection, asthenia, dizziness, emotional distress, lower respiratory infection and oropharyngeal pain. The incidence rate of AEs was significantly higher in the subgroup with ACs than in those without ACs for both PAI (5.2 vs 2.9 ACs per patient-year; incidence rate ratio (95% CI) 1.79 (1.54–2.08), *P*< 0.001) and SAI (3.41 vs 1.49 per patient-year; incidence rate ratio (95% CI) 2.29 (1.88–2.79), *P*< 0.001) (Table 5). Furthermore, the incidence rates for infectious intercurrent illnesses and infectious serious AEs (SAEs) were significantly higher in patients with ACs compared with those without ACs, both in PAI and SAI (Table 5). Importantly, the number of deaths was not different between the ACs and no ACs subgroups, however there were very few deaths in both the ACs and no ACs groups (Table 5).

**Table 5:** Incidence rate of important events based on patient-years. Patients with PAI and SAI who experienced ≥ 1 AC during the study period until the data cut-off point of February 5, 2019 were categorized as ‘≥ 1 AC’ (*n* = 84, *n* = 64, respectively). Those two groups were matched 1:3 for age, sex and AI type with patients with AI and no ACs, labelled ‘no AC’ (*n* = 252, *n* = 192, respectively).

	PAI				SAI			
	≥1 AC (*n* = 84)	No AC (*n* = 252)	IRR (95% CI)	*P* value	≥1 AC (*n* = 64)	No AC (*n* = 192)	IRR (95% CI)	*P* value
Any adverse event	5.2	2.9	1.8 (1.5–2.1)	<0.001	3.4	1.5	2.3 (1.9-2.8)	<0.001
Adrenal crisis	0.7	0	–	–	0.6	0	–	–
Infectious intercurrent illness	1.4	1.1	1.3 (1.1–1.5)	0.006	1.0	0.5	2.0 (1.5–2.5)	<0.001
Infectious serious adverse event	0.1	0.04	2.5 (1.4–4.2)	0.001	0.3	0.03	8.9 (4.7–17.0)	<0.001
Death	0	0.01	0*	–	0.01	0.01	1.4 (0.3–6.2)*	0.702
* n* (%)	0 (0)	6(2.4)	–	–	3 (4.7)	4 (2.1)	–	–

Incidence rate ratio (IRR) = number of reports per recorded patient-year; 95% CI is Exact Fisher 95% CI, *P* value from Fisher Exact Test; *For death the odds ratio with 95% CI based on patient-years is given.

AC, adrenal crisis; AI, adrenal insufficiency; PAI, primary AI; SAI, secondary AI.

## Discussion

This matched analysis of risk factors for ACs was based on the largest cohort of patients with AI enrolled in a prospective study, EU-AIR. This analysis showed an incidence of 6.53 prospectively recorded ACs per 100 patient-years in patients with PAI, and 3.17 per 100 patient-years in patients with SAI. In previous retrospective studies, the incidence of ACs has been reported to be 5.2–17 per 100 patient-years in PAI, and 3.6–5.8 per 100 patient-years in SAI (13, 14, 15). The only other prospective study to date reported an AC incidence of 8.3 per 100 patient-years in patients with AI, with ACs being fatal in 6.3% of cases (16).

The lower incidence of ACs in our study could be explained by the fact that the centres involved in EU-AIR are highly specialized tertiary endocrine centres, which offer frequent teaching for their patients (22) and equip them with AC emergency kits and steroid cards (3, 23, 24). Data from health insurance databases have shown a higher incidence of ACs than we report here (15, 25). This may be because in insurance databases many patients with AI will have been treated by family physicians or non-specialists, as opposed to endocrinologists, and will therefore not have received specialist training (26, 27) or be equipped to recognize or manage an AC (12, 28). In a recent US study, which reported a much higher incidence of 24 ACs per 100 patient-years, the authors highlighted possible reasons for the different AC rates found across studies (29). Important differences included the definition of AC used (for example, solely relying on a patient’s understanding of an AC), documentation of ACs by hospital report or by patients’ recall, and prospective or retrospective character of the study in question. It is also possible that the higher incidence reported in the US study could be due to more severe AI in the US cohort, the low patient questionnaire response rate of 35% causing a selection bias (29), or insufficient insurance coverage in the United States, in contrast to European health systems in which there is nearly 100% health insurance coverage.

As shown in a cross-sectional, questionnaire study by Hahner * et al.*, a high proportion of patients with AI experience ACs, some even reporting four or more ACs; however, a significant number of patients with PAI or SAI may never experience an AC (13). These results can be confirmed by our study, in which the vast majority of patients (94.5%) did not experience any ACs over the duration of the registry. In contrast, some patients were prone to experiencing repeated ACs, sometimes very frequently. For example, in patients with PAI who experienced an AC during the study period, 16.5% experienced > 1 AC per year; the corresponding number in patients with SAI was 9.4%. This raises the question of whether there is a specific type of patient with AI who is more prone to ACs. Therefore, in this study we characterized patients with AI who experienced ACs and compared them with patients without ACs.

We demonstrated that the occurrence of ACs in patients with PAI and SAI enrolled in EU-AIR is associated with differences in clinical and biochemical variables such as higher serum potassium and lower sodium levels, and lower diastolic blood pressure. In accordance with the results of studies by Hahner * et al.* (13) and White and Arlt (17), we found that in patients with PAI, presence of hypothyroidism was not associated with occurrence of ACs. However, in contrast to the study by Hahner * et al.*, in our study diabetes insipidus was not associated with ACs in patients with SAI (13).

In a retrospective analysis of the medical records of Dutch patients with AI, 35% of patients had experienced one or two ACs since diagnosis, whereas 7% had reported > 2 ACs (14). Data from the present prospective study provide more detailed insight into the incidence of ACs. In the Dutch study, risk factors for ACs were identified as cardiac, neurological or pulmonary comorbidities, and malignancy (14). Furthermore, the authors of that study found that the presence of other associated autoimmune diseases, such as diabetes mellitus type 1, thyroid disease, coeliac disease, vitiligo or autoimmune premature menopause, did not increase the risk of AC in a subgroup of patients with autoimmune PAI. We confirmed the findings that ACs were not associated with diabetes mellitus type 1, hypothyroidism or premature ovarian failure in PAI. This is in contrast to the study by White and Arlt, who reported that diabetes, asthma and premature ovarian failure were risk factors for AC (17). We did not investigate asthma in our study; however, one of the strengths of our analysis is that we matched patients with ACs for age and sex in the respective AI groups, which has not been performed in previous studies. In addition to examining the prevalence of concomitant diseases, we also examined the prevalence of traditional cardiovascular risk factors, given the increased vascular mortality in patients with AI. In our study we were not able to show an association between classical cardiovascular risk factors such as hypertension, diabetes mellitus, obesity or hyperlipidaemia with a risk profile for ACs, suggesting that these two processes – AC and vascular mortality – are likely independent of each other.

Our data show that patients with ACs have significantly more AEs than patients with no ACs. Furthermore, we analysed the incidence rates of infectious intercurrent illnesses and infectious SAEs based on the number of patient-years. Interestingly, in both PAI and SAI cohorts, patients with ACs showed a significantly higher incidence rate ratio for infectious events than patients with no ACs; in patients with SAI the incidence rate ratio was nearly doubled. Precipitating factors for ACs have been investigated by several groups; their results indicated that stressful conditions that lead to increased glucocorticoid demand, such as infectious disease, trauma, surgery, psychological distress and strenuous physical activity, increase the risk of ACs (11, 13, 16, 17). Therefore, it is thought that a higher risk for infectious diseases may be associated with AI and might lead to more frequent ACs (5, 11, 30). These findings can be corroborated by our study. It has also been shown that AI is associated with significantly decreased natural killer cell cytotoxicity, thereby potentially compromising early recognition and elimination of virally infected cells (31). Furthermore, the study by Isidori * et al.* indicated that patients with AI receiving conventional glucocorticoid replacement therapy multiple times a day exhibit a pro-inflammatory state and weakened immune defence (32).

However, importantly, the frequency of death was not different between the AC group and the no AC group in patients with either PAI or SAI. This is in accordance with previously published data from EU-AIR, which show that in patients who had experienced an AC, the cause of death was often not a result of the AC itself (33). Nevertheless, it is possible that the number of deaths within our matched cohort was too small to examine this endpoint fully.

We also found that patients with SAI with ACs were receiving a higher hydrocortisone dose and a higher proportion were receiving DHEA therapy than patients with SAI without ACs. It is known that the immune system is highly sensitive to glucocorticoids and that higher or multiple glucocorticoid doses may induce a more severe immune derangement. Therefore, it might be possible that those additional hydrocortisone doses could lead to the observed higher incidence rates of infectious events and ACs. Another possible explanation is that physicians may increase the dose of hydrocortisone and initiate DHEA therapy in response to patients feeling unwell, weak or lethargic. However, we did not include intermediate visits in our analysis to prove this assumption. A similar observation was noted in a cross-sectional study investigating the health-related quality of life (HRQoL) in patients with AI, which showed that patients who were receiving higher daily glucocorticoid doses displayed lower HRQoL (34). Although the data provided in EU-AIR were unable to pinpoint the cause of illness in these patients further, they support the current recommendation that lowering the dose of glucocorticoid replacement (within the recommended regimen) does not increase the likelihood of having an AC (35).

Interestingly, we found that potassium levels were significantly higher, but sodium levels were significantly lower, in patients with PAI with frequent ACs than in those without ACs, which indicates the possibility of inadequate mineralocorticoid replacement. This was found despite no difference in glucocorticoid doses per body weight or fludrocortisone doses between those with and without ACs. Blood pressure also tended to be lower in the frequent AC group, but only reached significance for diastolic blood pressure at last observation. The latter confirms a recent analysis showing that titration of the mineralocorticoid dose affects potassium levels but not blood pressure or fluid replacement (36). This suggests that patients with PAI with frequent ACs may be more vulnerable in situations that cause hypotension or a decrease in blood pressure, for example, infections with fever and pronounced sweating. A recent study showed that premature cardiovascular mortality in patients with PAI might result from high glucocorticoid replacement doses as well as high mineralocorticoid replacement doses (9). Therefore, in patients with PAI, fludrocortisone doses should not be too high, but importantly, insufficient levels that result in decreased sodium and increased potassium levels should be avoided (37).

This study has provided insights into the risk factors for ACs in patients with AI. A key limitation was the restricted number of clinical and biochemical variables that could be evaluated based on EU-AIR data. For patients with PAI, for example, data on renin were not available owing to the lack of standardisation of the assay across the study centres, and the fact that the value of renin measurement remains highly controversial (36). A further limitation is the fact that we did not have data on medications and doses regarding the treatment of hypogonadism, hypothyroidism or growth hormone deficiency in patients with SAI. Unfortunately, we were not able to differentiate SAI due to pituitary disease and SAI due to glucocorticoid-induced AI. It is also important to note that EU-AIR study centres are tertiary endocrine centres, which are specialized in treating patients with AI, hence our data may not be generalizable to all patients with AI. Furthermore, additional variables that were not measured in this study could be confounding factors. Finally, we did not have robust data on the number of ACs patients experienced before study entry and which previous interventions may have been undertaken to reduce future events, thereby introducing some bias to our data. Nevertheless, the strengths of the study include the use of real-world data, the prospective nature of the registry, and the large size of the cohorts, which were disease specific and matched for age and sex; this has not been the case in previous studies.

This matched analysis of risk factors for ACs based on the largest cohort of patients with AI enrolled in a prospective study demonstrated that the occurrence of ACs in patients with PAI and SAI is associated with higher serum potassium and lower sodium levels, and lower diastolic blood pressure, and that concomitant diseases and cardiovascular risk factors do not feature in the risk profile of AC. However, patients with ACs showed a higher incidence rate of infectious events, which could suggest an impaired immune system. Healthcare professionals should be aware that patients with frequent ACs may be more vulnerable in situations that can cause hypotension or a decrease in blood pressure, such as infections with fever and pronounced sweating.

## Declaration of interest

M Q, R D M, A M I and B E have received honoraria for talks and consultancy from ViroPharma and Shire, a member of the Takeda group of companies. A M I has also received consultancies, grants and personal support from Ipsen, Pfizer, Novo Nordisk and Corcept. C M is an employee of Shire International GmbH, a member of the Takeda group of companies. P Z is an employee of Shire Human Genetic Therapies, a member of the Takeda group of companies. R P is an employee of Baxalta Innovations GmbH, a member of the Takeda group of companies. A M Isidori is on the editorial board of EJE. A M Isidori was not involved in the review or editorial process for this paper, on which he is listed as an author.

## Funding

EU-AIR is funded by Shire Human Genetic Therapies Inc., a member of the Takeda group of companies. This research was funded by Shire, a member of the Takeda group of companies. Clinical Trial Information: Registration number NCT01661387.
